# Phase I and II randomized clinical trial of an oral therapeutic vaccine targeting human papillomavirus for treatment of cervical intraepithelial neoplasia 2 and 3

**DOI:** 10.1093/jncics/pkad101

**Published:** 2023-11-24

**Authors:** Kei Kawana, Osamu Kobayashi, Yuji Ikeda, Hideaki Yahata, Takashi Iwata, Toyomi Satoh, Azusa Akiyama, Daichi Maeda, Yumiko Hori-Hirose, Yukari Uemura, Kaori Nakayama-Hosoya, Kanoko Katoh, Yuki Katoh, Takahiro Nakajima, Ayumi Taguchi, Atsushi Komatsu, Mikiko Asai-Sato, Naoko Tomita, Kiyoko Kato, Daisuke Aoki, Shizunobu Igimi, Ai Kawana-Tachikawa, Danny J Schust

**Affiliations:** Department of Obstetrics and Gynecology, Nihon University School of Medicine, Tokyo, Japan; Department of Obstetrics and Gynecology, Nihon University School of Medicine, Tokyo, Japan; Department of Obstetrics and Gynecology, Nihon University School of Medicine, Tokyo, Japan; Department of Obstetrics and Gynecology, Graduate School of Medical Sciences, Kyushu University, Fukuoka, Japan; Department of Obstetrics and Gynecology, Keio University School of Medicine, Tokyo, Japan; Department of Obstetrics and Gynecology, Faculty of Medicine, University of Tsukuba, Ibaraki, Japan; Department of Obstetrics and Gynecology, Faculty of Medicine, University of Tsukuba, Ibaraki, Japan; Department of Molecular and Cellular Pathology, Graduate School of Medical Sciences, Kanazawa University, Ishikawa, Japan; Department of Central Laboratory and Surgical Pathology, National Hospital Organization Osaka National Hospital, Osaka, Japan; Department of Data Science, Center for Clinical Science, National Center for Global Health and Medicine, Tokyo, Japan; AIDS Research Center, National Institute of Infectious Diseases, Tokyo, Japan; Department of Obstetrics and Gynecology, Nihon University School of Medicine, Tokyo, Japan; Department of Functional Morphology, Nihon University School of Medicine, Tokyo, Japan; Department of Obstetrics and Gynecology, Nihon University School of Medicine, Tokyo, Japan; Laboratory of Human Single Cell Immunology, World Premier International Immunology Frontier Research Center, Osaka, Japan; Department of Obstetrics and Gynecology, Nihon University School of Medicine, Tokyo, Japan; Department of Obstetrics and Gynecology, Nihon University School of Medicine, Tokyo, Japan; Department of Obstetrics and Gynecology, Nihon University School of Medicine, Tokyo, Japan; Department of Obstetrics and Gynecology, Graduate School of Medical Sciences, Kyushu University, Fukuoka, Japan; Department of Obstetrics and Gynecology, Keio University School of Medicine, Tokyo, Japan; Department of Applied Biology and Chemistry, Tokyo University of Agriculture, Tokyo, Japan; AIDS Research Center, National Institute of Infectious Diseases, Tokyo, Japan; Department of Obstetrics and Gynecology, Duke University, Durham, NC, USA

## Abstract

**Background:**

Although many human papillomavirus (HPV)–targeted therapeutic vaccines have been examined for efficacy in clinical trials, none have been translated into clinical use. These previous agents were mostly administered by intramuscular or subcutaneous injection to induce systemic immunity. We investigated the safety and therapeutic efficacy of an HPV-16 E7-expressing *lacticaseibacillus*-based oral vaccine.

**Methods:**

In a double-blind, placebo-controlled, randomized trial, a total of 165 patients with HPV-16–positive high-grade cervical intraepithelial neoplasia 2 and 3 were assigned to orally administered placebo or low, intermediate, or high doses of IGMKK16E7 (*lacticaseibacillus paracasei* expressing cell surface, full-length HPV-16 E7). In the 4 groups, IGMKK16E7 or placebo was administered orally at weeks 1, 2, 4, and 8 postenrollment. The primary outcomes included histopathological regression and IGMKK16E7 safety.

**Results:**

In per-protocol analyses, histopathological regression to normal (complete response) occurred in 13 (31.7%) of 41 high-dose recipients and in 5 (12.5%) of 40 placebo recipients (rate difference = 19.2, 95% confidence interval [CI] = 0.5 to 37.8). In patients positive for HPV-16 only, the clinical response rate was 40.0% (12 of 30) in high-dose recipients and 11.5% (3 of 26) in recipients of placebo (rate difference = 28.5, 95% CI = 4.3 to 50.0). There was no difference in adverse events that occurred in the high-dose and placebo groups (*P* = .83). The number of HPV-16 E7–specific interferon-γ producing cells within peripheral blood increased with level of response (stable disease, partial, and complete responses; *P* = .004). The regression to normal (complete response) rates among recipients with high levels of immune response were increased in a dose-dependent manner.

**Conclusion:**

This trial demonstrates safety of IGMKK16E7 and its efficacy against HPV-16–positive cervical intraepithelial neoplasia 2 and 3. IGMKK16E7 is the first oral immunotherapeutic vaccine to show antineoplastic effects.

**Trial registration:**

jRCT2031190034.

Although cervical cancer risk has decreased in response to prophylactic human papillomavirus (HPV) vaccines, many low- and middle-income countries, especially in Eastern Mediterranean, Southeast Asian, and Western Pacific regions, lack robust implementation of prophylactic vaccination, and need for additional HPV-targeting therapies remains urgent (1-3). Because the precancerous lesion of cervical cancer, cervical intraepithelial neoplasia 2 and 3 (CIN 2/3), results from persistent infection with high-risk HPV, a therapeutic vaccine that induces host immunity against HPV is a promising treatment. Surgery, the only existing effective treatment for CIN 2/3, increases the risk of preterm delivery in current and subsequent pregnancies (4) and can also be challenging to implement in some low- and middle-income countries because of the lack of medical resources. Therefore, a noninvasive HPV-targeted therapeutic for CIN 2/3 is an unmet clinical need. Although many therapeutic HPV vaccines have been examined for efficacy in clinical trials (5-8), none have been translated into clinical use. The majority of prior therapeutic HPV vaccines induce systemic Th1 cellular immunity against HPV E6 and/or E7 by intramuscular or subcutaneous injection, and most therapeutic vaccines in development administer nucleic acids as expression vectors for HPV-16 and -18 E6 and/or E7 (5-7). Two double-blind, placebo-controlled, randomized clinical trials of therapeutic vaccination have been recently reported (9,10). The rate of clinical regression to normal is the critical clinical efficacy endpoint of a therapeutic agent for CIN; the VGX-3100 trial showed a complete regression rate of 40.2% (vs 16.7% in placebo) for CIN 2/3 associated with HPV-16 and -18 in post hoc analyses (9), whereas the Tipapkinogen Sovacivec vaccine trial showed a complete regression rate of 18.2% (vs 3.7% in placebo) (10) for CIN 2/3 associated with HPV-16.

Because CIN 2/3 is a cervical mucosal lesion, we hypothesized that induction of mucosal Th1 cellular immunity against HPV would be more effective for immunological elimination of CIN 2/3 than induction of systemic immunity. Several studies have demonstrated that integrin β7^+^ CCR9^+^ T cells involved in mucosal immunity home to the cervical mucosa (11-13). Because patients with CIN 2/3 often have difficulty in inducing cellular immunity against HPV in the cervical mucosa (14-16), we wanted to leverage the mucosal immune system to target HPV and developed an oral vaccination strategy using the intestinal mucosa, the most favorable site for induction of mucosal immunity (17,18). *Lacticaseibacillus paracasei* is a commonly ingested and safe lactic acid–producing microbe known to promote Th1 responses that target antigens delivered by the bacteria (19,20). We have generated *L paracasei* that express full-length HPV-16 E7 on their surface (21). In our 2 previous clinical trials, GLBL101c, another HPV-16 E7–expressing *L paracasei* species, was used and was effective in inducing regression of CIN 3 to CIN 2 or less (22) but demonstrated no clear efficacy against CIN 2 (23). In a phase I and IIa clinical study by the Korian study group, another HPV-16 E7–expressing *L paracasei* species, BLS-M07, was used, and the safety of oral vaccination and increases in serum HPV-16 E7–specific antibodies was demonstrated (24).

To improve the efficacy of this vaccination strategy, we have optimized surface expression of HPV-16 E7 on *L paracasei* to improve the induction of mucosal immunity in the intestine. We then formulated this HPV-16 E7–expressing *L paracasei* bacteria as an oral vaccine (IGMKK16E7) (25). Because oral therapeutic HPV vaccines are noninvasive treatments, one major advantage of oral administration is the minimization of medical resource usage, making these preferred products for use in low- and middle-income countries.

Here, we conducted a phase I and /II double-blind, placebo-controlled, randomized clinical trial in which 165 patients with HPV-16–positive CIN 2/3 were administered oral IGMKKK16E7 or placebo. We assessed IGMKK16E7 vaccine safety and therapeutic efficacy against HPV-16–positive CIN 2/3 as measured by clinical lesion regression.

## Methods

### Trial oversight

The mucosal immunotherapy using HPV type 16 E7–expressing *Lactobacillus*-based vaccine for the treatment of cervical high-grade squamous intraepithelial lesion study (MILACLE study) was an investigator-initiated multicenter, randomized, double-blinded, placebo-controlled clinical trial conducted at 4 centers in Japan (Japan Registry Clinical Trial ID: jRCT2031190034). The trial design has been published previously (26). The clinical trial was supported by grants from the Japan Agency for Medical Research and Development and GLOVACC Co Ltd; the funding agencies had no influence on study design or trial implementation and were not involved in data collection or analysis, in the writing of the manuscript, or in the decision to submit it for publication. The trial protocol was approved by the individual institutional review boards of each study site (No. 3004-1505 by Nihon University on 2019/3/29; 2018313 by Kyushu University on 2019/3/23; D18-03 by Keio University on 2019/4/5; and I-32 by Tsukuba University on 2019/3/28) and was overseen by a Japanese governmental pharmaceuticals and medical devices agency (PMDA No. 30-4536 on December 7, 2018). The trial was performed in accordance with the principles of the Declaration of Helsinki. All authors assume responsibility for the accuracy and completeness of the data and analyses, as well as for the fidelity of the trial and this report to the protocol.

### Patients

Patients with pathologically determined CIN 2 or 3 who were positive for HPV-16 by HPV genotyping using exfoliated cervical cells were eligible for enrollment. A central review for pathological diagnosis was set up to exclude bias in pathology diagnosis between the 4 centers and among pathologists. Two pathologists specializing in gynecologic oncology independently observed whole slide images obtained from biopsies taken under colposcopy by gynecologic oncologists and determined the pathological diagnosis by consensus. Registration was based on the results of the central pathological review. HPV typing was performed using a commercial testing company; positive for HPV-16 only or positive for HPV-16 and other types were differentiated and recorded. Patients aged 20-45 years were included. Pregnant or postpartum women were excluded. Patients with allergies to lactic acid beverages or dairy products were excluded. Patients with findings suggestive of invasive cancer were also excluded. A complete list of the inclusion and exclusion criteria is provided in the Clinical Trial Protocol (Supplementary Materials, available online). All participants provided written informed consent.

### Trial procedures

Participants were randomly assigned in a 1:1:1:1 ratio to either the low-dose, intermediate-dose, or high-dose IGMKK16E7 groups or the placebo group. We calculated sample size based on the results of the GLBL-101c clinical study targeting CIN 3 (22). The response rate for CIN 3 (expected value) in this clinical trial was assumed to be as follows: 10%, 20%, 30%, and 40% for the placebo, low-dose, intermediate-dose, and high-dose groups, respectively. For CIN 3 patients, the minimum number of patients needed to exceed the detection power of 80% was calculated using the Cochran–Armitage test at 5% statistically. Based on the assumption that the dropout rate would be approximately 10%, the number of patients needed per group was calculated to be 31 patients. To explore the effectiveness of IGMKK16E7 against CIN 2, the number of CIN 2 patients was set to 10 patients. Therefore, the target number of patients in this clinical trial was set to 41 patients for each of the 4 groups, totaling 164 patients.

Random assignment was performed with the use of a web-based system by minimization (dynamic balancing) and was stratified according to center, CIN grading (CIN 2 or 3), and HPV genotype (HPV-16 only or HPV-16 and other types). All patients received 4 rounds of oral immunization at weeks 1, 2, 4, and 8. Low-dose (0.5 g/day), intermediate-dose (1.0 g/day), or high-dose (1.5 g/day) IGMKK16E7 or placebo were administered orally after fasting once each morning for 5 days during each treatment week. IGMKK16E7 is an attenuated *L paracasei* displaying a genetic fusion of mutated full-length HPV-16 E7 protein to an anchoring protein derived from the lactic acid bacteria on the bacterial cell surface with optimized efficiency (25). Heat-attenuated IGMKK16E7 powder was orally administered and encapsulated in a seamless capsule, and dosage choice for low, intermediate or high-dose exposures to IGMKK16E7 was determined based on our previous studies (see Clinical Trial Protocol in the Supplementary Materials, available online) (22,23). Patients were followed without surgical intervention (including biopsy) until 16 weeks after the first dose. Pathological evaluation (colposcopy-directed biopsy) was performed 16 weeks after the first dose to avoid missing patients who had progressed to invasive cancer; if the lesion was CIN 2 or lower at the 16-week timepoint, the patient was again evaluated pathologically 24 weeks after the first dose. If a patient was diagnosed clinically as CIN 3 or more at week 16, the patient received standard therapy. Pathological diagnosis of all biopsy specimens was performed centrally by 2 independent pathologists in accordance with World Health Organization pathological criteria (27).

### Clinical samples and biological assays

Cervices were sampled before registration and at 16 and 24 weeks after the first study dose in outpatient clinics. Peripheral blood for immunological evaluation and exfoliated cervical cells for cytological and virological evaluation were sampled at study weeks 9, 16, and 24. Peripheral blood monocytes were isolated within several hours after blood sampling, frozen gently, and stored at −80ºC until bulk analysis. Exfoliated cervical cells for virological evaluation were transferred to a central laboratory. Viral status was assayed using the PCR-rSSO (Polymerase chain reaction-reverse sequence specific oligonuclotide) HPV genotyping method (Luminex, Belgium). For immunological analyses, peripheral blood monocytes were cultured with HPV-16 E7 overlapping peptides for 2 weeks in the presence of interleukin-7 and -15 to expand HPV-16 E7–specific T cells (28). HPV-16 E7–specific IFN-γ producing cells in the expanded peripheral blood monocytes sampled preimmunization, and at weeks 16 and 24, were quantitated by enzyme-linked immunosorbent spot assay using HPV-16 E7 overlapping peptides as a stimulant.

### Outcomes

Primary outcomes included histopathological regression to normal (complete response) or CIN 1 (partial response) at week 16 and IGMKK16E7 safety. Secondary outcomes included histopathological regression at week 24, immunological response, cytological regression, and viral clearance. All safety outcomes were based on adverse event reporting (see the Clinical Trial Protocol for criteria, see the Supplementary Materials).

### Statistical analysis

The study was designed to have 80% power to detect a 30%, 20%, and 10% difference in the rates of pathological complete regression or complete regression plus partial regression between high-, intermediate-, low-dose, and placebo groups, respectively. Because spontaneous regression to normal or CIN 1 with placebo was estimated at 10%, at least 12 outcome events were required in the high-dose group for the study to be conclusive; we aimed to include 40 patients for each group (26). Outcomes for histopathological regression were analyzed by Fisher exact testing, and potential dose response patterns among the groups were assessed. Immunological data were log-transformed and compared by analysis of covariance or trend testing among the groups. Prespecified subgroup analyses of the primary outcome were performed for the variables shown in Table 1. All analyses were performed using full- and per-protocol sets. Two-sided *P* values of no more than .05 were considered statistically significant. Analyses were performed with SAS software, version 9.4 (SAS Institute) and R software, version 3.3.1 (R Project for Statistical Computing).

**Table 1.** Baseline demographics and other characteristics in patients of full analysis set^a^

Statistic or Category	Placebo	IGMKK16E7			Total
		Low dose	Intermediate dose	High dose	
No. of Patients	40	41	40	43	164
Age, y					
Mean (SD)	34.1 (5.0)	33.9 (4.8)	34.1 (5.3)	34.2 (5.0)	34.1 (4.9)
Median (IQR)	34.0 (23-43)	33.0 (26-44)	32.0 (27-44)	34.0 (23-44)	34.0 (23-44)
Height, cm					
Mean (SD)	158.3 (4.7)	160.3 (5.1)	159.0 (4.7)	159.0 (5.0)	159.2 (4.9)
Median (IQR)	157.9 (150-167)	159.4 (150-170)	159.1 (150-168)	159.2 (150-171)	159.2 (150-171)
Body weight, kg					
Mean (SD)	55.1 (9.5)	53.0 (6.1)	55.9 (9.6)	55.4 (8.6)	54.8 (8.6)
Median (IQR)	52.1 (41-75)	53.4 (42-70)	54.1 (41-95)	54.5 (42-88)	54.0 (41-95)
Pathological diagnosis					
CIN 2	9 (22.5)	10 (24.4)	9 (22.5)	11 (25.6)	39 (23.8)
CIN 3	31 (77.5)	31 (75.6)	31 (77.5)	32 (74.4)	125 (76.2)
HPV genotype at baseline					
HPV-16 only	26 (65.0)	29 (70.7)	27 (67.5)	30 (69.8)	112 (68.3)
HPV-16 and other subtypes	14 (35.0)	12 (29.3)	13 (32.5)	13 (30.2)	52 (31.7)

a CIN = cervical intraepithelial neoplasia; HPV = human papillomavirus; HSIL = high-grade squamous intraepithelial lesion; IQR = interquartile range.

## Results

### Characteristics of the patients

**Figure 1.**
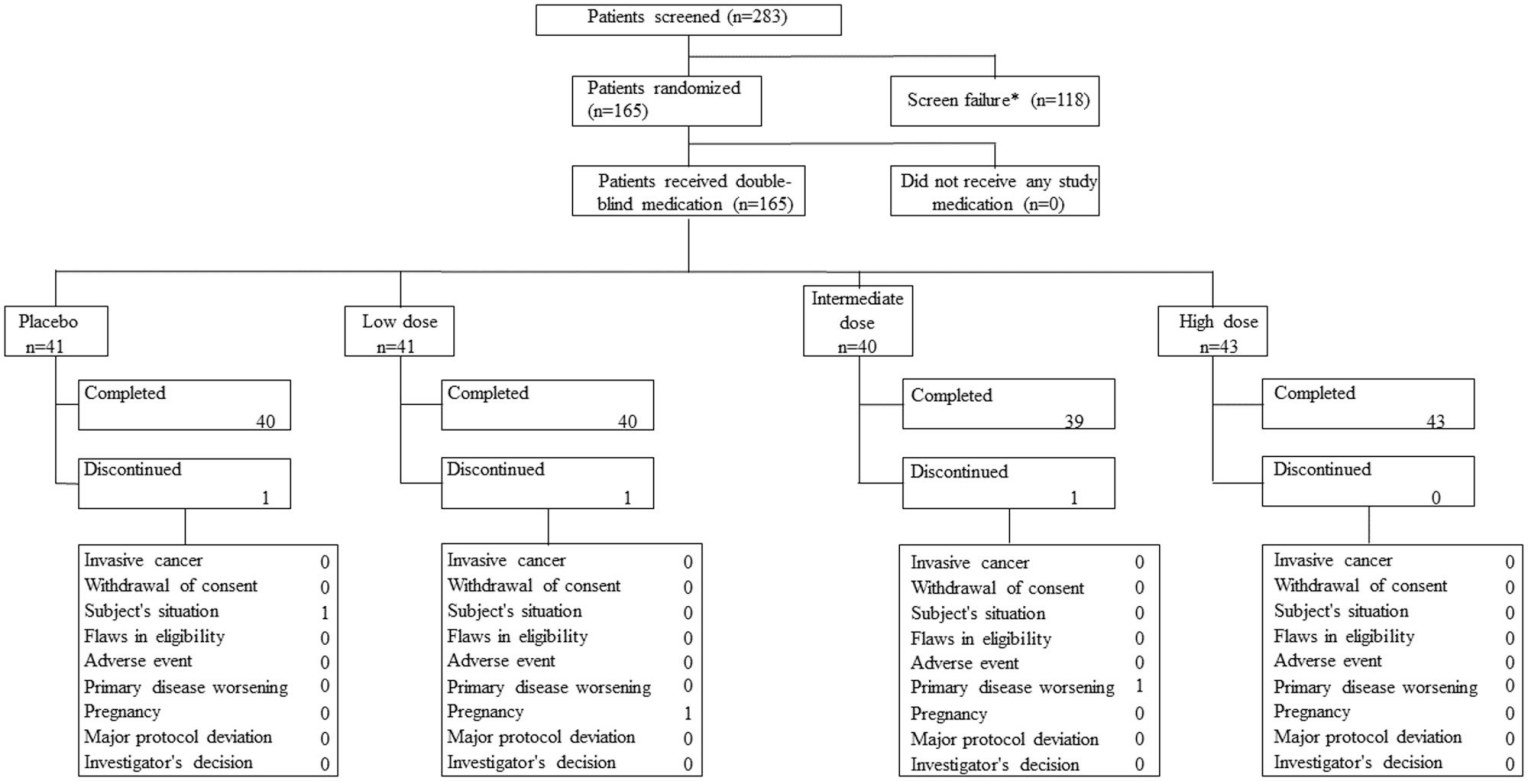
Patients’ enrollment and random assignment. Screening exclusions at registration included women who were human papillomavirus-16 negative and those not diagnosed with cervical intraepithelial neoplasia 2 or 3 by central pathology (*). The remaining 165 patients were enrolled and participated in a double-blind random assignment. A total of 165 patients were assigned to all groups, and 3 patients were discontinued from the study.

From June 15, 2019, to December 30, 2021, a total of 283 patients were enrolled at 4 centers; 165 eligible patients were randomly assigned to the high-dose (n = 43), intermediate-dose (n = 40), and low-dose (n = 41) groups, and 40 patients were assigned to the placebo group for the full analysis set (Figure 1). The 4 groups were balanced using minimization, and baseline characteristics did not differ among the groups (Table 1). In the full analysis set, 39 and 125 patients were diagnosed as CIN 2 and CIN 3, respectively. Nearly every patient received target doses of IGMKK16E7 or placebo according to the protocol. Only 1 patient per group had a study drug compliance rate of less than 80%. Overall, 68.3% (112 of 164) of patients with CIN 2/3 were positive for HPV-16 only, and the remaining 52 (31.7%) patients were positive for HPV-16 and other HPV genotypes. A total of 37 (22.6%) patients (placebo: 8 [20%]; low dose: 7 [17%]; intermediate dose: 10 [25%]; high dose: 12 [28%]) were diagnosed with CIN 3 or more at week 16 and received standard therapy. No patients had progression to invasive cancer during the study.

### Clinical outcome

**Figure 2.**
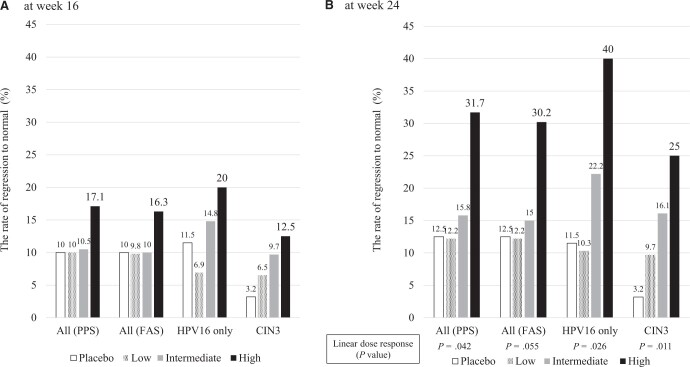
Rate of regression to normal (complete response). **A)** The regression to normal (complete response) rates at 16 weeks after the first dose for each treatment group is shown for all participants (full analysis set, per-protocol set) and subgroups of patients with human papillomavirus (HPV)-16–only positive results and cervical intraepithelial neoplasia (CIN) 3. **B)** The complete response rates at 24 weeks after the first dose for each treatment group are shown. A linear dose response in complete response rates is indicated using *P* values, with a linear dose response observed in patients with HPV-16 subtype only or with CIN 3 at baseline. FAS = full analysis set; PPS = per-protocol set.

In per-protocol set analysis of 159 patients, histopathological regression to normal (complete response) occurred in 7 (17.1%) of 41 high-dose recipients and 4 (10.0%) of 40 placebo recipients at week 16 (rate difference = 7.1, 95% confidence interval [CI] = −9.4 to 23.9), while complete response occurred in 13 (31.7%) of 41 high-dose and 5 (12.5%) of 40 placebo recipients at week 24 (rate difference = 19.2, 95% CI = 0.5 to 37.8) (Figure 2;Supplementary Table 1, available online). In the full analysis set of 164 patients, complete response occurred in 13 (30.2%) of 43 high-dose, 7 (15.0%) of 40 intermediate-dose, 5 (12.2%) of 41 low-dose, and 5 (12.5%) of 40 placebo recipients (rate difference between high dose and placebo = 17.7, 95% CI = −0.4 to 35.6) (Figure 2, B;Supplementary Table 1, available online). In the full analysis set of 112 patient with CIN 2/3 associated with HPV-16 only, complete response occurred in 12 (40.0%) of 30 high-dose, 6 (22.2%) of 27 intermediate-dose, 3 (10.3%) of 29 low-dose, and 3 (11.5%) of 26 placebo recipients; the complete response rate in the high-dose group was statistically greater than with placebo (rate difference = 28.5, 95% CI = 4.3 to 50.0) (Figure 2, B;Supplementary Table 1, available online). In the full analysis set of 125 patients with CIN 3 at baseline, complete response occurred in 8 (25.0%) of 32 high-dose, 5 (16.1%) of 31 intermediate-dose, 3 (9.7%) of 31 low-dose, and 1 (3.2%) of 31 placebo recipients. The complete response rate in the high-dose group was significantly higher than with placebo (rate difference = 21.8, 95% CI = 4.4 to 40.3) (Figure 2, B;Supplementary Table 1, available online). A linear complete response rate dose response was demonstrated for IGMKK16E7 in overall per-protocol set analysis (*P* = .042), full analysis set subgroup analysis of patients positive for HPV-16 only (*P* = .026), and the full analysis set subgroup of patients with CIN 3 at baseline (*P* = .01) using the maximum contrast method (Figure 2, B). Although the complete response plus partial response rates were higher than that of complete response alone in IGMKK16E7 and placebo recipients, the difference in complete response plus partial response rates between high-dose and placebo groups was not statistically different, and the linear dose response for IGMKK16E7 was obscured in complete response plus partial response rates (Supplementary Figure 1 and Supplementary Table 1, available online).

**Figure 3.**
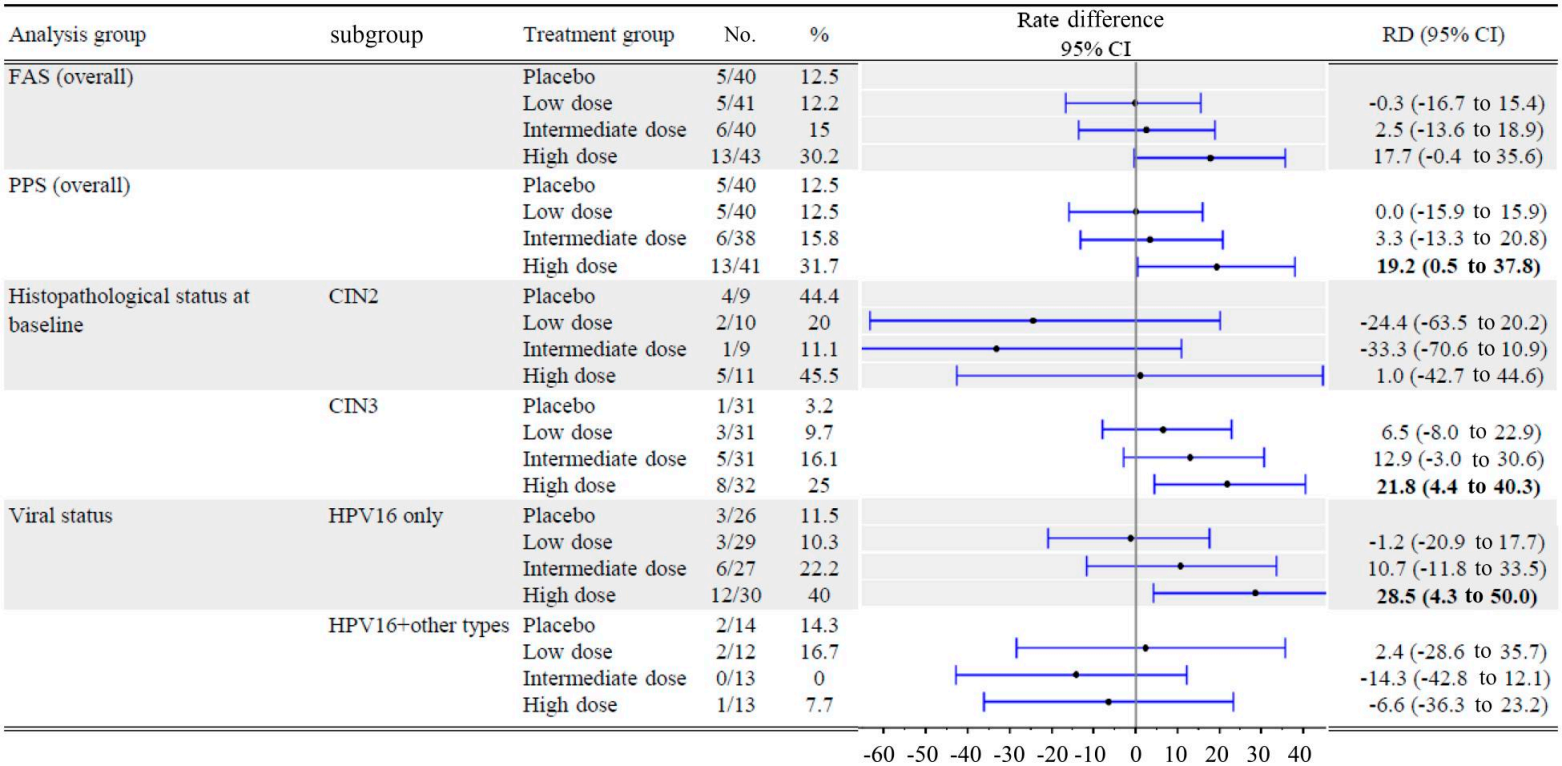
Forest plots of differences in complete response rates for vaccine compared with placebo treatments in various subgroups. Rate differences and their 95% confidence intervals are shown in forest plots for complete response rate comparisons of various treatment vs placebo groups 24 weeks after the first vaccine dose. Patients with human papillomavirus (HPV)-16 and other HPV subtypes or cervical intraepithelial neoplasia 2 were small in number (approximately 10 patients), and complete response rates did not differ between treatment groups. CI = confidence interval; CIN = cervical intraepithelial neoplasia; FAS = full analysis set; HPV = human papillomavirus; PPS = per-protocol set; RD = rate difference. Bold means statistical difference.

A forest plot analysis of differences in complete response rates between treatment and placebo for various subgroups is shown in Figure 3. The 95% confidence intervals for rate differences as represented beside the forest plots demonstrate clear statistical difference in high-dose recipients with CIN 3 at baseline and in solely HPV-16–positive patients. Neither viral clearance nor cytological regression were associated with clinical efficacy or drug groups (Supplementary Table 2, available online).

### Immunological analysis

**Figure 4.**
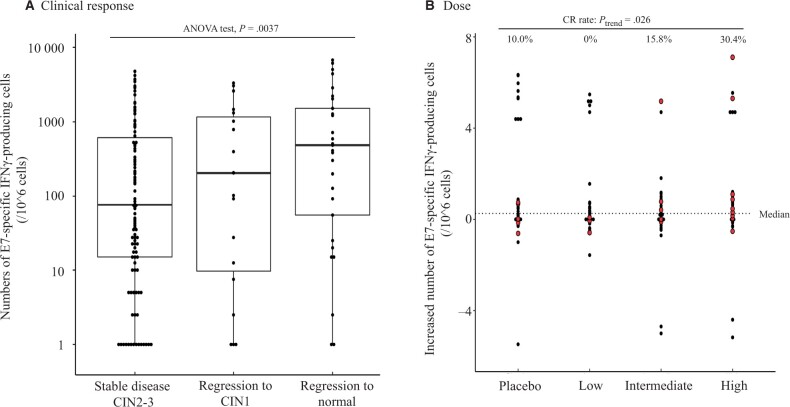
Immunological responses and clinical efficacy. To evaluate the magnitude of human papillomavirus (HPV)-16 E7–specific Th1 immune response, the numbers of HPV-16 E7–specific interferon-γ–producing cells in peripheral blood were obtained by enzyme-linked immunosorbent spot assay. The spot-forming cell numbers (spot-forming cells per 10^6 peripheral blood monocytes) for each patient were plotted on the *y* axis. **A)** Comparisons of spot-forming cells at the study endpoint among regression to normal, regression to cervical intraepithelial neoplasia (CIN) 1, and stable disease (CIN 2-3) groups. **B)** Change in spot-forming cells for each patient, grouped by treatment type and dose. **Red dots** depict patients with complete response, and the **dotted line** represents the median of spot-forming cell change among the full analysis set. ANOVA = analysis of variance; CR = complete response; IFN = interferon.

To examine whether clinical efficacy could be linked to immunological response, we assessed the association between clinical response and the number of HPV-16 E7–specific, IFN-γ–producing cells (spot-forming cells numbers) among peripheral blood monocytes at the study endpoint. The measured spot-forming cells among peripheral blood monocytes collected at the study endpoint increased in parallel to clinical disease regression status (stable disease, partial response, and complete response; analysis of variance test, *P* = .004) (Figure 4, A). Spot-forming cells in the complete response group were clearly higher than that of non–complete response group (*P* = .005) (Supplementary Table 3, available online). We did not, however, detect any dose dependency for spot-forming cells measured at the study conclusion (Supplementary Figure 2, available online). To account for differences in individual overall host immune responsiveness, we reanalyzed our data on a per-patient basis, measuring spot-forming cell changes before and after vaccine administration (Figure 4, B). In Figure 4, B, changes in spot-forming cells (before and at treatment completion) for each participant were plotted on the *y* axis grouped by treatment. Red dots depict patients with complete response, and the dotted line represents the median change in spot-forming cells among full analysis set. Complete response rates among recipients with higher than median spot-forming cell changes (above the dotted line) increased in a dose-dependent manner (*P*_trend_ = .026).

### Safety

In the safety analysis set of 164 patients, at least 1 adverse event occurred in 21 (48.8%) of 43 high-dose, 21 (52.5%) of 40 intermediate-dose, 20 (48.8%) of 41 low-dose, and 19 (46.3%) of 41 placebo recipients questioned 24 weeks after the first dose (percentage point difference between high dose and placebo = 2.5, 95% CI = −0.19 to 0.24; *P* = .83) (Supplementary Table 4, available online). High-grade events (grade 3) occurred in only 2 low-dose recipients (Supplementary Table 4, available online), and neither serious events nor events leading to treatment withdrawal occurred. Gastrointestinal disorders, including abdominal pain, occurred in 11 (25.6%) patients receiving high-dose vaccine and 8 (19.5%) receiving placebo (rate difference = 6.1, 95% CI = −0.13 to 0.25). Gynecological disorders, including vaginal discharge, occurred in 1 (2.3%) high-dose and 2 (4.9%) placebo recipients (rate difference = −2.6, 95% CI = −0.15 to 0.08). Other adverse events, including mild allergic symptoms, were uncommon and occurred in all groups (Supplementary Table 4, available online).

## Discussion

We found that oral administration of IGMKK16E7 in patients with HPV-16–positive CIN 2 and 3 drove histopathological regression to normal (complete response) at 24 weeks after the first dose. Linear complete response dose responses for IGMKK16E7 were clearly demonstrated in both overall and subgroup analyses. Differences in the complete response rate between high-dose and placebo groups appeared to diverge by 24 weeks, but not by 16 weeks, after the first dose. In our safety analysis set, adverse events did not occur in the IGMKK16E7 recipients at a higher incidence than that in placebo recipients. More than 50% of IGMKK16E7 recipients experienced no adverse events. These observations show IGMKK16E7 to be less invasive and to be characterized by an improved safety profile compared with injectable vaccinations.

Particularly novel in this clinical trial was the use of the intestinal mucosa for induction of cellular immune responses against CIN 2/3 through oral vaccine administration. Notably, our double-blind, randomized trial demonstrated that complete response rates increase in a linear dose-dependent manner, strongly supporting that histopathological regression to normal is a pharmacological effect of IGMKK16E7. There are no previously reported randomized clinical trials of therapeutic HPV vaccines that demonstrate dose-dependent histopathological regression of precancerous cervical disease. IGMKK16E7 is the first HPV therapeutic oral vaccine with efficacy comparable with that of the best parenteral vaccine so far evaluated in placebo-controlled efficacy trials (9,10). HPV therapeutic oral vaccines have advantage over conventional intramuscular and subcutaneous vaccines in that they are a noninvasive treatment. Another major advantage of the oral vaccine is that it can be administered with minimal consumption of medical resources, making it particularly useful in low- and middle-income countries. This might even be extended to mass immunization of adult women in the absence of HPV testing or other screening in such settings.

Complete response, but not complete response plus partial response, efficacy was detected in comparisons of IGMKK16E7 and placebo recipients. Regression to normal seems to require a more stringent induction of antigen-specific immune response than regression to CIN 1, but when considering potential for use as a therapeutic agent, combined pathological regression to normal and viral clearance is the most favorable parameter to evaluate the clinical efficacy of HPV therapeutic vaccine. Because there was no stastical difference between vaccine and placebo for viral clearance in this trial, further improvement of IGMKK16E7 is desired to achieve the therapeutic effect of viral clearance. Indeed, partial response occurred more frequently in placebo and low-dose (15% and 17%, respectively) than in intermediate- and high-dose recipients (5% and 9%, respectively). Spontaneous regression was noted in all treatment groups. This is not surprising, especially in patients with CIN 2, and can even occasionally occur in patients with CIN 3 (29). Spontaneous regression may mask or overestimate the efficacy of a therapeutic vaccine and highlights the importance of conducting a double-blind, placebo-controlled randomized trial.

Results from immunological analyses support a pharmacological effect of IGMKK16E7. Higher numbers of circulating E7-specific, IFN-γ–producing cells (spot-forming cells) in patients with histopathological improvement is consistent with the involvement of an E7-specific Th1 response in histopathological lesion regression. The number of spot-forming cells measured at the endpoint was not IGMKK16E7 dose dependent; however, spot-forming cells at baseline varied widely among recipients, and the measured spot-forming cells after administration may have been affected not only by the pharmacological effect of vaccine but also by baseline spot-forming cells. To account for this possibility, the change in spot-forming cells before and after treatment observed in patients with complete response was represented by treatment group. In the full analysis set, the complete response rate of those recipients with a spot-forming cell change higher than the median value (hyper-responders) increased in a dose-dependent manner. Of note, some of the placebo recipients were also hyper-responders. It may not be surprising that some patients had a preexisting E7-specific T-cell population in their peripheral blood monocytes that was present but not fully effective because they had persistent HPV-16 infection. These cells would also be expanded by the E7 stimulation used in our experimental immunologic assay design. However, unlike hyper-responders in the high-dose group who exhibited a higher level of complete response than those in the placebo group, none of the hyper-responsive individuals who had received placebo have a complete response, suggesting their E7-specific T cells may not have had antineoplastic activity against CIN 2 or 3. IGMKK16E7-induced E7-specific T cells may acquire and exert more favorable antineoplastic characteristics. Further immunological analysis of the specific characteristics of these 2 sets of E7-specific T cells will be addressed in future studies. A phase III IGMKK16E7 clinical trial will be conducted in the future.

Our efficacy and safety data highlight the therapeutic promise of orally administered IGMKK16E7 as a safe, noninvasive, and effective nonsurgical intervention for patients with high-grade precancerous cervical lesions.

## Supplementary Material

pkad101_Supplementary_Data

## Data Availability

Participant data can be shared with outside collaborators for research to understand more about the clinical efficacy and safety of the IGMKK16E7 and immune response to the vaccine obtained from the MILACLE study. These data are available online at https://www.nichidaisanfujinka.com/milaclestudy.
