# Supplementary material for: Phase I and II randomized clinical trial of an oral therapeutic vaccine targeting human papillomavirus for treatment of cervical intraepithelial neoplasia 2 and 3
Source: JNCI Cancer Spectr. 2023 Nov 24;7(6):pkad101. doi: 10.1093/jncics/pkad101 (PMC10748578; doi:10.1093/jncics/pkad101)
Supplement: pkad101_Supplementary_Data [file pkad101_supplementary_data.pdf]

Supplementary tables

Supplementary Table 1

Supplementary Table 2

Supplementary Table 3

Supplementary Table 4

Supplementary Table 1 Summary of clinical efficacy of IGMKK16E7

	Placebo		Low dose		Intermediate dose		High dose	
Number of subjects in full analysis set (FAS)	40		41		40		43	
Number of subjects in per-protocol set (PPS)	40		40		38		41	
Number of subjects with CIN3 at baseline in FAS	31		31		31		32	
Number of subjects with CIN2 at baseline in FAS	9		10		9		11	
Number of subjects with HPV16 only in FAS	26		29		27		30	
Number of subjects with HPV16+other types in FAS	14		12		13		13	
Regression to normal (CR)	n (%)							
At 16w in FAS	4	(10.0)	4	(9.8)	4	(10.0)	7	(16.3)
At 16w in PPS	4	(10.0)	4	(10.0)	4	(10.5)	7	(17.1)
At 16w in patients with CIN3 at baseline	1	(3.2)	2	(6.5)	3	(9.7)	4	(12.5)
At 16w in patients with CIN2 at baseline	3	(33.3)	2	(20.0)	1	(11.1)	3	(27.3)
At 16w in patients with HPV16 only	3	(11.5)	2	(6.9)	4	(14.8)	6	(20.0)
At 16w in patients with HPV16+other types	1	(7.1)	2	(16.7)	0	(0.0)	1	(7.7)
At 24w in FAS	5	(12.5)	5	(12.2)	6	(15.0)	13	(30.2)

At 24w in PPS	5	(12.5)	5	(12.5)	6	(15.8)	13	(31.7)
At 24w in patients with CIN3 at baseline	1	(3.2)	3	(9.7)	5	(16.1)	8	(25.0)
At 24w in patients with CIN2 at baseline	4	(44.4)	2	(20.0)	1	(11.1)	5	(45.5)
At 24w in patients with HPV16 only	3	(11.5)	3	(10.3)	6	(22.2)	12	(40.0)
At 24w in patients with HPV16+other types	2	(14.3)	2	(16.7)	0	(0.0)	1	(7.7)
Regression to normal/CIN1 (CR+PR)	n (%)							
At 16w in FAS	8	(20.0)	9	(22.0)	6	(15.0)	12	(27.9)
At 16w in PPS	8	(20.0)	9	(22.5)	6	(15.8)	12	(29.3)
At 16w in patients with CIN3 at baseline	4	(12.9)	5	(16.1)	4	(12.9)	7	(21.9)
At 16w in patients with CIN2 at baseline	4	(44.4)	4	(40.0)	2	(22.2)	5	(45.5)
At 16w in patients with HPV16 only	5	(19.2)	6	(20.7)	5	(18.5)	9	(30.0)
At 16w in patients with HPV16+other types	3	(21.4)	3	(25.0)	1	(7.7)	3	(23.1)
At 24w in FAS	11	(27.5)	12	(29.3)	8	(20.0)	17	(39.5)
At 24w in PPS	11	(27.5)	12	(30.0)	8	(21.1)	17	(41.5)
At 24w in patients with CIN3 at baseline	5	(16.1)	8	(25.8)	6	(19.4)	10	(31.3)
At 24w in patients with CIN2 at baseline	6	(66.7)	4	(40.0)	2	(22.2)	7	(63.6)

At 24w in patients with HPV16 only	7	(26.9)	9	(31.0)	6	(22.2)	13	(43.3)
At 24w in patients with HPV16+other types	4	(28.6)	3	(25.0)	2	(15.4)	4	(30.8)

Supplementary Table 2 Viral clearance and cytological regression in FAS analyses

FAS analyses	Groups							
	Placebo		Low dose		Intermediate dose		High dose	
Viral clearance	n / total case no. (%)							
HPV16-negative at 16w	0/40	(0.0)	1/40	(2.5)	3/40	(7.5)	2/43	(4.7)
HPV16-negative at 24w	2/40	(5.0)	4/40	(10.0)	4/40	(10.0)	3/43	(7.0)
Cytological regression at 16w	n / total case no. (%)							
Regression to LSIL, ASC-US, NILM	8/30	(26.7)	8/36	(22.2)	6/32	(18.8)	4/34	(11.8)
Stable diseases (HSIL)	20/30	(66.7)	24/36	(66.7)	23/32	(71.9)	28/34	(82.4)
Progression (SCC)	0/30	(0.0)	0/36	(0.0)	0/32	(0.0)	0/34	(0.0)
Cytological regression at 24w	n / total case no. (%)							
Regression to LSIL, ASC-US, NILM	7/30	(23.3)	8/36	(22.2)	6/32	(18.8)	10/34	(29.4)
Stable diseases (HSIL)	15/30	(50.0)	18/36	(50.0)	15/32	(46.9)	10/34	(29.4)
Progression (SCC)	0/30	(0.0)	0/36	(0.0)	0/32	(0.0)	0/34	(0.0)
Unknown [a)]	8/30	(26.7)	10/36	(27.8)	11/32	(34.4)	14	(41.2)

a) "Unknown" includes "not evaluable" and missing of judgement due to no sampling or any other reason.

Supplementary Table 3 Relation between Histopathological Diagnossis Result s and SFC Value of HPV E7-Specific Immune Response

SFC / 10^6 cells [a]	CR group	Non-CR group
Number of subjects in FAS	29	129
n	29	87
mean	1125.60	419.45
SD	1741.53	880.65
minimum	0.0	0.0
median	385.00	45.00
maximum	6320.0	4752.5
95% CL_lower	463.16	231.76
95% CL upper	1788.05	607.15
Group Comparison		
Mean difference (Group1-Group2)		706.15
95% CL_lower		216.28
95% CL_upper		1196.02
P value		0.0051

a) Measurement by ELISpot assay using PBMCs sampled until 24 weeks after the first dose

Supplementary Table 4 Adverse events with onset until 24 weeks after the first dose

	n (%) a)							
	Placebo		Low dose		Intermediate dose		High dose	
Number of subjects in safety analysis set (N)	41		41		40		43	
Number of subjects at least one event occurred	19 (46.3)		20 (48.8)		21 (52.5)		21 (48.8)	
Number of subjects with high-grade (grade 3 to 5 in NCI-CTCAE) event occurred	0 (0.0)		2 (4.9)		0 (0.0)		0 (0.0)	
Blood and lymphatic system disorders	0	(-)	1	(2.4)	0	(-)	0	(-)
Lymphadenopathy	0	(-)	1	(2.4)	0	(-)	0	(-)
Cardiac disorders	0	(-)	1	(2.4)	0	(-)	0	(-)
Palpitations	0	(-)	1	(2.4)	0	(-)	0	(-)
Eye disorders	0	(-)	0	(-)	0	(-)	1	(2.3)
Retinal haemorrhage	0	(-)	0	(-)	0	(-)	1	(2.3)
Gastrointestinal disorders	8	(19.5)	12	(29.3)	8	(20.0)	11	(25.6)
Abdominal distension	1	(2.4)	0	(-)	0	(-)	0	(-)
Abdominal pain	0	(-)	2	(4.9)	2	(5.0)	1	(2.3)
Abdominal pain upper	4	(9.8)	1	(2.4)	1	(2.5)	5	(11.6)
Constipation	3	(7.3)	0	(-)	0	(-)	1	(2.3)
Diarrhoea	0	(-)	5	(12.2)	2	(5.0)	2	(4.7)
Gastrointestinal disorder	1	(2.4)	0	(-)	0	(-)	0	(-)
Mouth ulceration	0	(-)	0	(-)	1	(2.5)	0	(-)
Nausea	3	(7.3)	4	(9.8)	2	(5.0)	5	(11.6)

Stomatitis	0	(-)	1	(2.4)	0	(-)	1	(2.3)
General disorders and administration site conditions	0	(-)	4	(9.8)	3	(7.5)	3	(7.0)
Chest pain	0	(-)	1	(2.4)	0	(-)	0	(-)
Discharge	0	(-)	0	(-)	0	(-)	1	(2.3)
Peripheral swelling	0	(-)	0	(-)	0	(-)	1	(2.3)
Pyrexia	0	(-)	3	(7.3)	3	(7.5)	1	(2.3)
Hepatobiliary disorders	0	(-)	1	(2.4)	0	(-)	0	(-)
Hepatic function abnormal	0	(-)	1	(2.4)	0	(-)	0	(-)
Infections and infestations	7	(17.1)	1	(2.4)	6	(15.0)	7	(16.3)
Gastroenteritis	1	(2.4)	0	(-)	0	(-)	1	(2.3)
Influenza	0	(-)	1	(2.4)	0	(-)	0	(-)
Nasopharyngitis	3	(7.3)	1	(2.4)	3	(7.5)	6	(14.0)
Rhinitis	0	(-)	0	(-)	0	(-)	1	(2.3)
Tonsillitis	0	(-)	0	(-)	2	(5.0)	0	(-)
Upper respiratory tract infection	2	(4.9)	0	(-)	0	(-)	0	(-)
Vulvovaginal candidiasis	1	(2.4)	0	(-)	1	(2.5)	0	(-)
Injury, poisoning and procedural complications	0	(-)	0	(-)	1	(2.5)	0	(-)
Contusion	0	(-)	0	(-)	1	(2.5)	0	(-)
Investigations	2	(4.9)	4	(9.8)	0	(-)	0	(-)
Alanine aminotransferase increased	0	(-)	1	(2.4)	0	(-)	0	(-)
Blood triglycerides increased	1	(2.4)	1	(2.4)	0	(-)	0	(-)
Coronavirus test positive	0	(-)	1	(2.4)	0	(-)	0	(-)

Lymphocyte count decreased	1	(2.4)	1	(2.4)	0	(-)	0	(-)
Protein urine present	0	(-)	1	(2.4)	0	(-)	0	(-)
Metabolism and nutrition disorders	0	(-)	0	(-)	1	(2.5)	1	(2.3)
Hypertriglyceridaemia	0	(-)	0	(-)	1	(2.5)	1	(2.3)
Musculoskeletal and connective tissue disorders	0	(-)	0	(-)	1	(2.5)	2	(4.7)
Back pain	0	(-)	0	(-)	0	(-)	1	(2.3)
Intervertebral disc protrusion	0	(-)	0	(-)	0	(-)	1	(2.3)
Tenosynovitis	0	(-)	0	(-)	1	(2.5)	0	(-)
Nervous system disorders	6	(14.6)	9	(22.0)	6	(15.0)	6	(14.0)
Dizziness	0	(-)	0	(-)	1	(2.5)	0	(-)
Headache	4	(9.8)	8	(19.5)	5	(12.5)	5	(11.6)
Hypoaesthesia	0	(-)	0	(-)	0	(-)	1	(2.3)
Mental impairment	0	(-)	0	(-)	0	(-)	1	(2.3)
Migraine	1	(2.4)	0	(-)	0	(-)	0	(-)
Restless legs syndrome	0	(-)	1	(2.4)	0	(-)	0	(-)
Somnolence	1	(2.4)	0	(-)	0	(-)	0	(-)
Psychiatric disorders	0	(-)	0	(-)	0	(-)	1	(2.3)
Panic disorder	0	(-)	0	(-)	0	(-)	1	(2.3)
Renal and urinary disorders	0	(-)	1	(2.4)	0	(-)	0	(-)
Pollakiuria	0	(-)	1	(2.4)	0	(-)	0	(-)
Reproductive system and breast disorders	2	(4.9)	2	(4.9)	1	(2.5)	1	(2.3)
Dysmenorrhoea	1	(2.4)	1	(2.4)	0	(-)	1	(2.3)
Uterine haemorrhage	0	(-)	0	(-)	1	(2.5)	0	(-)

Vaginal discharge	1	(2.4)	1	(2.4)	0	(-)	0	(-)
Respiratory, thoracic and mediastinal disorders	1	(2.4)	3	(7.3)	0	(-)	2	(4.7)
Dyspnoea	0	(-)	1	(2.4)	0	(-)	0	(-)
Laryngeal pain	1	(2.4)	0	(-)	0	(-)	0	(-)
Oropharyngeal pain	0	(-)	2	(4.9)	0	(-)	1	(2.3)
Rhinorrhoea	0	(-)	0	(-)	0	(-)	1	(2.3)
Skin and subcutaneous tissue disorders	2	(4.9)	2	(4.9)	2	(5.0)	3	(7.0)
Acne	0	(-)	0	(-)	1	(2.5)	0	(-)
Dermatitis	0	(-)	0	(-)	0	(-)	1	(2.3)
Dry skin	0	(-)	1	(2.4)	0	(-)	0	(-)
Eczema	1	(2.4)	1	(2.4)	0	(-)	0	(-)
Pruritus	0	(-)	0	(-)	1	(2.5)	2	(4.7)
Rash	1	(2.4)	0	(-)	0	(-)	0	(-)

a) n (%). Incidence proportion (%) = 100 (n/N). In case where multiple events classified into same SOC (or PT)

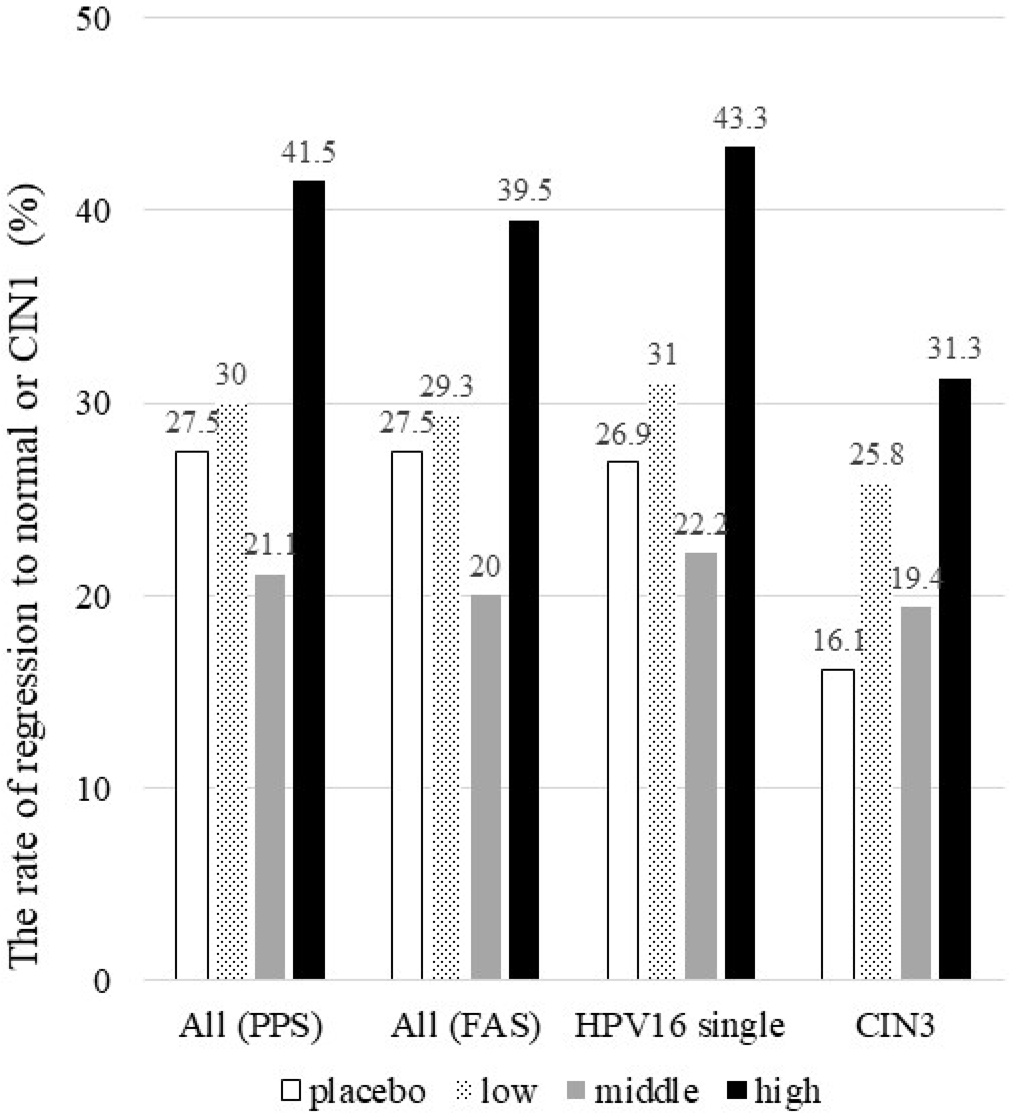

Supplementary Figure 1. Rate of regression to normal or CIN1 (CR+PR)

The CR+PR rates at 24 weeks after the first dose for each treatment group are shown for the combined subject groups (FAS, PPS) and subgroups of patients positive for HPV16 only and those with CIN3 (Supplementary Table1).

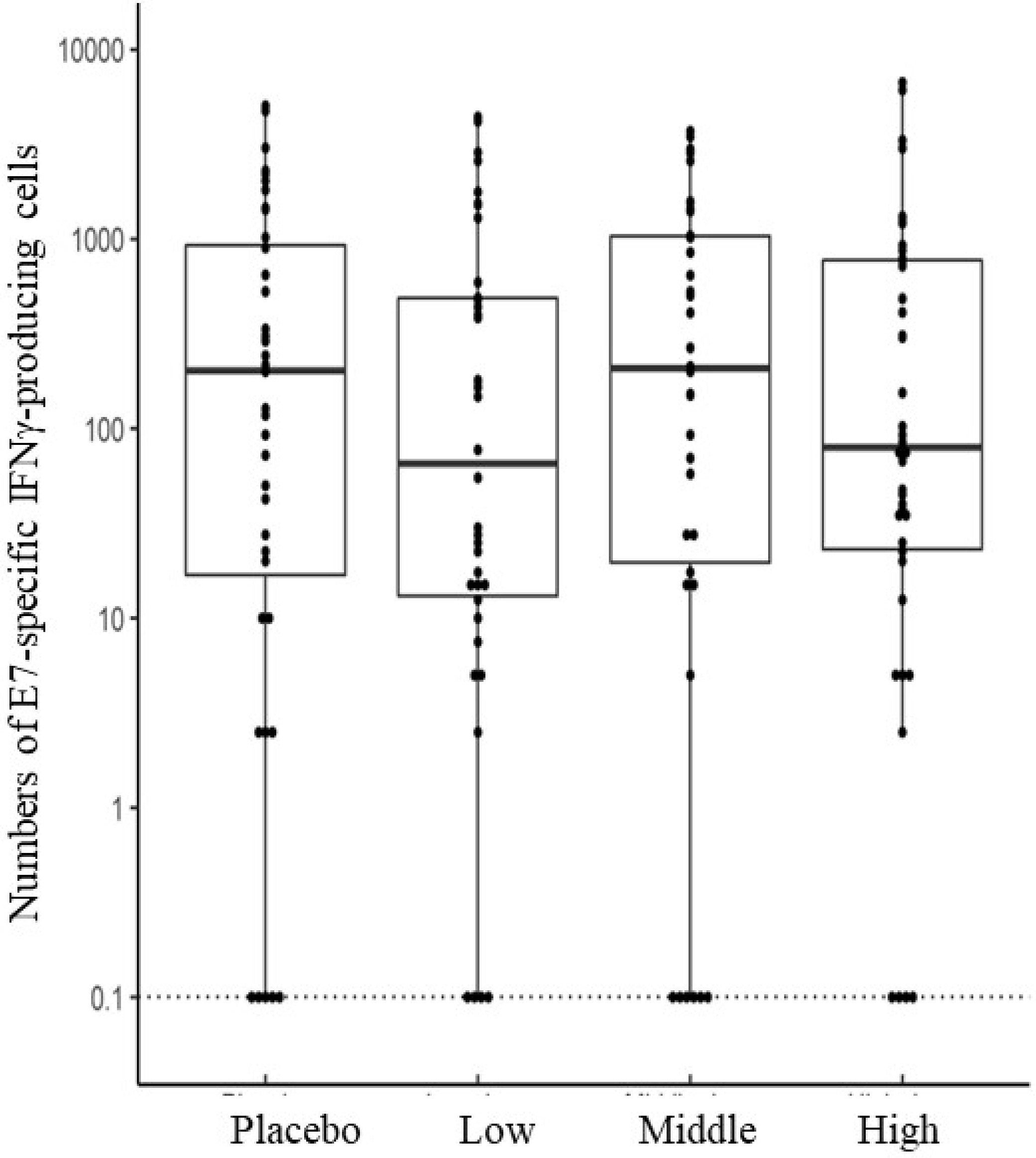

Supplementary Figure2. Immunological responses for each treatment dose group

The numbers of HPV16 E7-specific IFNγ-producing cells in peripheral blood were obtained by ELISPOT assay. The spot-forming cell numbers (SFC / 10^6 PBMCs) for each patient were plotted on the Y axis for each treatment dose group.
